# Great leap forward famine exposure and urban-rural migration convolute the modern prevalence of diabetes in China

**DOI:** 10.1186/s41043-024-00596-2

**Published:** 2024-07-30

**Authors:** Dian Luo, Wan-chin Kuo

**Affiliations:** 1 Department of Population Health Sciences, School of Medicine and Public Health, University of Wisconsin-Madison, Madison, WI USA; 2 School of Nursing, University of Wisconsin-Madison, Madison, WI USA

## Abstract

**Background:**

Although evidence from birth cohort analysis has indicated the metabolic risk of early-life exposure to the Great Leap Forward Famine (GLFF) in China, three confounding effects, including the exposure windows, aging, and geographical variations in famine severity, have been brought to debates for a decade. This study aimed to address these confounding effects and extensively examine how GLFF exposure is associated with diabetes risk in mid-to-late life and its interaction with urban-rural migration.

**Methods:**

Data from the China Health and Retirement Longitudinal Study (CHARLS) were analyzed with age-stratification and stepped wedge approaches. Weighted prevalence and multivariable logistic regression were used to investigate the effects of GLFF exposure and urban-rural migration on mid-to-late life diabetes risk and the interaction between GLFF exposure and urban-rural migration. Birth provinces were controlled as a fixed effect to account for variations in famine severity across provinces.

**Results:**

Compared to those who were never exposed to GLFF, fetal GLFF exposure was associated with a higher risk of adult-onset diabetes after controlling for provinces, demographics, and health statuses. Yet, after adding the proxy of childhood growth environments into the model, fetal exposure to GLFF was not significantly associated with adult-onset diabetes risk (OR = 1.22, *p* = 0.10), compared to those who were never exposed to GLFF. Across the three age-stratification groups, static urban residents, in general, had a higher risk of diabetes compared to static rural residents. Interaction effects between GLFF exposure and urban-rural migration were insignificant across all three age-stratification groups.

**Conclusion:**

Fetal exposure to GLFF might have a traceable effect on adult-onset diabetes risk. Yet, the growth environment and urban lifestyle outweigh and further confound the impact of GLFF exposure on adult-onset diabetes risk.

**Supplementary Information:**

The online version contains supplementary material available at 10.1186/s41043-024-00596-2.

## Significance

**What is already known on this topic:**

Although evidence from the birth-cohort approach indicates that fetal exposure to the Great Leap Forward Famine (GLFF) was associated with an increased risk of diabetes in mid-to-late life, three major critics have been brought forward in the debate, including exposure window, aging, and geographic confounding effects.

**What this study adds:**

This study addressed the confounding effects in debate and delineated the convolution of GLFF exposure and urban-rural migration in the current prevalence of diabetes in China. Findings underscore the profound effect of urban-rural migration on diabetes risk in mid-to-late life.

**How this study might affect research, practice or policy:**

This study highlights the notion that diabetes has multifaceted origins, shaped by immediate and intermediate environments (urban/rural/migration statuses and childhood growth environment) and broader cultural and historical contexts (GLFF exposure). Current findings inform practice and policy development in three areas: (1) urban food quality is more important than food quantity, across the maternal and childhood development, when gauging midlife diabetes risk, (2) continuous update in modern food standards is warranted, and (3) smart food choices, health education, and preventive care services are needed to complement current food policies.

**Supplementary Information:**

The online version contains supplementary material available at 10.1186/s41043-024-00596-2.

## Introduction

Fetal famine exposure substantially increases the risk of cardiovascular diseases, metabolic syndrome, and diabetes in mid-to-late life [1–3]. The Fetal Origins Hypothesis has been observed in many war-time-related famine events, including civil wars, genocides, and World War II (WWII). The most well-known study is the Dutch Hunger Winter Famine, which indicated that prenatal exposure to poor nutrition increased the lifetime risk of metabolic syndrome and diabetes [2, 3]. Similarly, the Ukraine Famine Study, the Pol Pot Era study, and the WWII studies had indicated an increased lifetime risk of diabetes in those birth cohorts exposed to war-time related famine events, compared to those who were unexposed to these events [4–8].

The Great Leap Forward was an economic and social campaign from 1958 to 1962 in China. Due to the lack of comprehensive and scientifically informed policies at that time, around 15 to 55 million people died of famine [9]. This campaign resulted in a non-wartime mass famine and hunger event in human history and has been documented as the Great Leap Forward Famine (GLFF). Similar to those historical famine events, recent studies in China show that those with fetal exposure to GLFF were 2-fold likely to have diabetes in mid-to-late life, compared to those who were not exposed to GLFF in their early life [10–14]. However, two methodological limitations have been brought forward in this line of research with the birth cohort approach: the confounding effects of exposure window and the age-related diseases and functional declines [15, 16]. Researchers concerning the confounding effects of exposure window found that the early-life exposure to GLFF was predominantly defined by fetal-famine exposure. They recommended further distinguishing the exposure window among adolescent exposure, childhood exposure, and fetal exposure [17–19]. These researchers grouped historical famine exposure based on birth years, such as defining adolescent exposure for those who were born in 1940–1947 and defining childhood exposure for those who were born in 1948–1957. However, a school of scientists raised concerns pertaining to the birth cohort approach, because this approach is subject to the confounding effect of the natural aging process. Specifically, the reduced telomere length, mitochondrial dysfunction, and altered body composition during the biological aging process could synergistically elevate individuals’ risk of cardiovascular diseases, diabetes, and comorbidities [15, 16]. Hence, they argue that the observed GLFF effects using birth cohort approach may be attributed to the natural aging process rather than distinct cohort exposure to GLFF [20]. Existing studies examining GLFF effects on disease prevalence have not yet reached a solution that accounts for both confounding effects of exposure window and the natural aging process.

In addition to the confounding effects of exposure window and aging, Garnaut and scientists have pointed out a central weakness in the research of central-local dynamics during GLFF [21]. New data with grain procurement maps have indicated that the urban areas, including the adjacent handicrafts, were not severely affected by famine during the GLFF. Garnaut and researchers in this field have urged the need to understand the rural-urban differences during the GLFF and rural-urban migration in China when equating the GLFF exposure to the modern prevalence of diabetes in China. Nevertheless, a recent systematic review and meta-analysis concluded that the effect of GLFF exposures on diabetes risk did not differ between rural and urban residents, based on findings from two studies [22]. In fact, Wang and colleagues found that those who were exposed to the GLFF and grew up in urban areas (defined by the gross domestic product per capita) had a higher risk of diabetes compared to those who were exposed to GLFF and grew up in the rural areas [14]. Such inconsistent findings in the body of literature might be explained by the binary approach of urban-rural statues, without further examination of urban-rural migration. We argue that internal migration concomitates urban-rural variances, leading to potential inflation in Type 2 Error; to minimize the concomitant effects, a distinction between static urban/rural residence and urban/rural migration is needed.

To address the gaps in the literature and the unsolved confounding effects in debates, we used age-stratification and stepped wedge approaches and operated urban-rural migration with four statuses. This study has two specific aims: (1) to examine the degree to which GLFF exposure is associated with diabetes risk in mid-to-late life, and (2) to examine whether urban-rural migration and migration statuses moderate the relationship between GLFF exposure and mid-to-late-life diabetes risk.

## Methods

### Dataset

This study utilized data from the China Health and Retirement Longitudinal Study (CHARLS), which offers insights into the socio-psychological, financial, and health aspects of middle-aged and older adults in mainland China. The sampling procedure for CHARLS began with the direct selection of county-level units across 28 of the 32 mainland provinces, excluding Tibet. To refine the selection further, the National Bureau of Statistics’ village-level data was employed to determine village and community units within these counties. As a result, 450 primary sampling units (PSUs) were established using a probability proportional-to-size sampling approach, which translated to three PSUs for each county. Households within each PSU were then pinpointed using maps derived from Google Earth. Ultimately, all identified households were approached to participate in the survey [23]. The CHARLS collected data in four waves from 2011 to 2018. The first wave, conducted between June 2011 and March 2012, sampled 17,708 individuals from 450 communities. During each subsequent wave, adults aged between 40 and 44, along with their partners, were invited to join the CHARLS as the refreshment sample [24].

The CHARLS was approved by the Institutional Review Board (IRB) at Peking University (IRB 00001052–11,014). This secondary data analysis was approved by the Social Sciences IRB at the University of Wisconsin-Madison with an exemption from full IRB review.

### Sample

In this study, we initially evaluated a cohort from the CHARLS dataset comprising 22,883 individuals. Our focused analysis, however, centered on 13,911 participants who provided complete information on diabetes status, birth cohort, and demographic details. To mitigate the confounding influence of age on diabetes risk assessment, we implemented age-stratification and stepped wedge methodologies. Table 1 illustrates our grouping strategy, where individuals from different survey waves were categorized to ensure comparable age ranges at the time of survey response. For instance, in Group 1, individuals exposed to famine during childhood and surveyed in 2018, aged 61–71, were paralleled with those exposed in adolescence and surveyed in 2011, aged 64–72. Group 2 compared fetal-exposed respondents from 2018 (aged 56–61) with childhood-exposed respondents from 2011 (aged 54–64). In Group 3, non-exposed participants from 2018 (aged 50–56) were matched with fetal-exposed participants from 2013 (aged 51–56). This methodological approach was instrumental in diminishing the impact of age-related confounding, enabling us to conduct a risk comparison across adjacent famine exposure groups.

**Table 1:** Age-stratification and stepped wedge approaches with the risk comparison between the adjacent exposure windows

Groups	GLFF exposure window and survey wave			
**Group 1** (Age 61–72)	Adolescence-exposed (Survey wave: 2011) *N* = 2,417	Childhood-exposed (Survey wave: 2018) *N* = 5,345	Fetal-exposed (Survey wave: not available)	Non-exposed (Survey wave: not available)
Group 1 Age Range during the Survey	64–72	61–71		
**Group 2** (Age 54–64)	Adolescence-exposed (Survey wave: not available)	Childhood-exposed (Survey wave: 2011) *N* = 5,575	Fetal-exposed (Survey wave: 2018) *N* = 2,193	Non-exposed (Survey wave: not available)
Group 2 Age Range during the Survey		54–64	56–61	
**Group 3** (Age 50–56)	Adolescence-exposed (Survey wave: not available)	Childhood-exposed (Survey wave: not available)	Fetal-exposed (Survey wave: 2013) *N* = 2,240	Non-exposed (Survey wave: 2018) *N* = 3,025
Group 3 Age Range during the Survey			51–56	50–56

### Dependent variable

The dependent variable is a binary variable indicating whether or not the participants have been diagnosed with diabetes, including any treatment, medication, or lifestyle modification for diabetes.

### Historical famine exposure

The primary independent variable is historical famine exposure. We identified four exposure cohorts according to their age of exposure to GLFF: (1) Individuals who were born between 1963 and 1967 as the non-exposed cohort; (2) individuals who were born between 1958 and 1962 as the fetal-exposed cohort; (3) individuals who were born between 1948 and 1957 as the childhood-exposed cohort; (4) individuals who were born between 1940 and 1947 as the adolescence-exposed cohort.

### Urban/rural/migration statuses

Rural-to-urban migration is defined as individuals who move from rural hukou to urban areas in order to work or live without changing hukou statuses [25]. Following Long and colleagues’ operational scheme, urban-rural migration status was identified based on participants’ current residential places and hukou records. Accordingly, participants were categorized into four groups: static urban residence, rural-to-urban migration, urban-to-rural migration, and static rural residence. Hukou identity is a unique household registration system tied to individuals’ birthplaces [26]. Individuals born in rural areas were assigned a rural hukou, while individuals born in urban areas were assigned an urban hukou. Once assigned, a hukou identity is tied into their household record [27]. Current residential places in CHARLS were classified based on the National Bureau of Statistics in China, which identifies a community as an urban area if it is in a city, suburb of a city, or place with more than 70% of non-agricultural workforce [28].

### Covariates

Sociodemographic factors (including age, sex, education, and marital status) and health risk factors (including self-reported health status, smoking status, drink status, and health insurance status) were included in the analysis as potential confounders influencing diabetes risk. Variables of guardian alcohol/drug issue, self-report childhood health, and self-report childhood finance were controlled as the proxy measures of childhood growth environments. Guardian alcohol/drug issue was obtained from a question asking participants whether their female guardian had alcohol/drug problems during the years they were growing up.

Due to the large number of individuals who retired, we used household consumption per capita instead of household income per capita to measure participants’ economic status. Household consumption per capita was operationalized as the total expenditures in the household (including food, rental/housing, clothing, communication expenses, utility, fuels for transportation, service expenditures, entertainment, daily necessities, and medical expenses) divided by numbers of people living in the household.

### Statistical analysis

Descriptive statistics were used to compare the socio-demographics and baseline health status among the three urban-rural migration statuses. Data analyses were built based on listwise deletion, under the assumption that missingness in covariates, except income, was missing at random (MAR). Data were analyzed using the survey procedures in Stata to account for the complex survey design in the CHARLS dataset.

Weighted prevalence and weighted multivariable logistic regression were used to examine the association between GLFF exposure and diabetes risk in mid-to-late life while controlling for the socio-demographics and health risk factors. Since the severity of famine was different in different provinces, we also included the birth provinces as the fixed effect in our model [21].

To compare the effects of GLFF exposure, urban-rural migration status, and interaction effects on diabetes risk in mid to late life, we compared three models: (1) GLFF exposure with covariates; (2) GLFF exposure and urban-rural migration status with covariates, (3) GLFF exposure, urban-rural migration status, as well as the interaction term (URBAN*GLFF) and covariates. The formulas of these four models are listed below, and $\:{\lambda\:}_{i}$ is the province fixed effect term:

1 $$Diabete{s_i} = \beta {\:_0} + \beta {\:_1}GLF{E_i} + \beta {\:_2}Covariate{s_i} + \lambda {\:_i} + {\varepsilon _i}$$

2 $$\begin{gathered} Diabete{s_i} = {\beta _0} + {\beta _i}GLF{E_i} + \hfill \\\,\,\,\,\,\,\,\,\,\,\,\,\,\,\,\,\,\,\,\,\,\,\,\,\,\,\,{\beta _2}Migration\,Statu{s_{_i}} + \hfill \\\,\,\,\,\,\,\,\,\,\,\,\,\,\,\,\,\,\,\,\,\,\,\,\,\,{\beta _3}Co\operatorname{var} iate{s_i} + {\lambda _i} + {\varepsilon _i} \hfill \\ \end{gathered}$$

3 $$\begin{gathered} Diabete{s_i} = {\beta _0} + {\beta _1}GLF{E_i} + \hfill \\\,\,\,\,\,\,\,\,\,\,\,\,\,\,\,\,\,\,\,\,\,\,\,\,{\beta _2}Migration\:Statu{s_i} + \hfill \\\,\,\,\,\,\,\,\,\,\,\,\,\,\,\,\,\,\,\,\,\,\,\,\,\,\,{\beta _3}GLF{E_i} \times Migration\:Statu{s_i} + \hfill \\\,\,\,\,\,\,\,\,\,\,\,\,\,\,\,\,\,\,\,\,\,\,\,\,{\beta _4}Covariate{s_i} + {\lambda _i} + {\varepsilon _i} \hfill \\ \end{gathered}$$

Margin estimation was used to examine the associations between GLFF exposure and diabetes risk moderated by urban/rural/migration statuses. All reported *p*-values were two-tailed, with *p*-values less than 0.05 considered significant. All the analyses were performed using Stata SE 17 (Stata Corp, College Station, USA).

## Results

### Missing data management

As shown in Supplement Tables 1 and 2, missing values were identified in ten covariates. The chi-square test of missing patterns in covariates with outcome variable (diagnosis of diabetes) did not reject the null hypothesis, except income variable. Therefore, in current study, listwise deletion was performed under the assumption that missingness in covariates, except income, was MAR. Since income variable was subject to missing not at random (MNAR) and was not the essential independent variable in the current study, we performed post hoc analysis by deleting income variable from the analysis. As shown in Supplement Tables 3, 4 and 5, deleting income variable from the model did not significantly change the results.

**Table 2:** Descriptive statistics

	2011 Adolescence-Exposed vs. 2018 Childhood-Exposed						2011 Childhood-Exposed vs. 2018 Fetal-Exposed						2013 Fetal-Exposed vs. 2018 Non-Exposed					
	2018 Childhood-Exposed			2011 Adolescence-Exposed			2018 Fetal-Exposed			2011 Childhood-Exposed			2018 Non-Exposed			2013 Fetal-Exposed		
Age	Mean	SD	Weighted Mean	Mean	SD	Weighted Mean	Mean	SD	Weighted Mean	Mean	SD	Weighted Mean	Mean	SD	Weighted Mean	Mean	SD	Weighted Mean
	65.25	0.04	65.26	67.15	0.05	67.26	58.09	0.03	58.11	58.31	0.04	58.29	53.24	0.03	53.23	53.12	0.03	53.12
**Diabetes prevalence**	**N**	**%**	**Weighted %**	**N**	**%**	**Weighted %**	**N**	**%**	**Weighted %**	**N**	**%**	**Weighted %**	**N**	**%**	**Weighted %**	**N**	**%**	**Weighted %**
Non-diabetes	3,939	82.8	82.5%	1,679	82.8	82.9%	1,650	85.4	84.8%	4,000	83.4	83.3%	2,314	88.2	88.6%	1,689	85.8	85.5%
Diabetes	816	17.2	17.5%	349	17.2	17.1%	283	14.6	15.2%	794	16.6	16.7%	310	11.8	11.4%	279	14.2	14.5%
**Migration Status**																		
Static urban residence	671	14.1	20.8%	313	15.4	21.5%	284	14.7	20.7%	693	14.5	21.3%	351	13.4	18.0%	294	14.9	20.9%
Rural-to-urban migration	944	19.9	21.8%	364	18.0	19.5%	437	22.6	24.2%	949	19.8	21.7%	601	22.9	23.6%	432	22.0	24.1%
Static rural residence	3140	66.0	57.4%	1351	66.6	58.9%	1212	62.7	55.1%	3152	65.8	57.1%	1,672	63.7	58.4%	1,242	63.1	55.0%
**Sex**																		
Male	2295	48.3	49.0%	1011	49.9	50.6%	922	47.7	49.3%	2342	48.9	49.9%	1,194	45.5	46.1%	933	47.4	48.3%
Female	2,460	51.7	51.0%	1,017	50.2	49.4%	1011	52.3	50.7%	2,452	51.2	50.1%	1,430	54.5	53.9%	1,035	52.6	51.7%
**Education level**																		
No formal education	1463	30.8	28.0%	687	33.9	31.4%	367	19.0	17.4%	1435	29.9	27.0%	291	11.1	10.1%	374	19.0	16.9%
Below elementary school	1,087	22.9	22.5%	380	18.7	18.2%	288	14.9	13.8%	1,124	23.5	23.0%	350	13.3	13.2%	293	14.9	14.4%
Elementary school	1,034	21.8	21.5%	577	28.5	28.8%	340	17.6	18.2%	1,038	21.7	21.1%	694	26.5	25.4%	322	16.4	16.6%
Middle school	788	16.6	18.6%	264	13.0	14.0%	551	28.5	28.6%	817	17.0	19.0%	933	35.6	34.8%	567	28.8	28.8%
High school	268	5.6	6.3%	36	1.8	2.6%	339	17.5	18.5%	264	5.5	6.2%	262	10.0	11.4%	359	18.2	19.7%
College and above	115	2.4	3.2%	84	4.1	5.2%	48	2.5	3.5%	116	2.4	3.7%	94	3.6	5.1%	53	2.7	3.7%
**Marital status**																		
Unsingle	4041	85.0	0.8490376	1409	69.5	0.6880335	1759	91.0	90.9%	4078	85.1	84.3%	2,468	94.1	93.8%	1,785	90.7	90.7%
Single	714	15.0	15.1%	619	30.5	31.2%	174	9.0	9.1%	716	14.9	15.7%	156	6.0	6.2%	183	9.3	9.3%
**Self-reported health status**																		
Good	396	8.3	8.4%	123	6.1	6.9%	230	11.9	13.7%	379	7.9	8.1%	381	14.5	14.7%	222	11.3	12.7%
Fair	2535	53.3	54.6%	931	45.9	48.2%	1034	53.5	53.0%	2523	52.6	54.0%	1,467	55.9	57.7%	1,053	53.5	54.0%
Poor	1,824	38.4	37.0%	974	48.0	44.9%	669	34.6	33.3%	1,892	39.5	38.0%	776	29.6	27.6%	693	35.2	33.2%
**Smoke status**																		
No	3042	64.0	64.0%	1305	64.4	65.2%	1227	63.5	62.5%	3060	63.8	63.3%	1,743	66.4	66.5%	1,260	64.0	63.8%
Yes	1,713	36.0	36.0%	723	35.7	34.8%	706	36.5	37.5%	1,734	36.2	36.7%	881	33.6	33.5%	708	36.0	36.2%
**Alcohol drink status**																		
No	2405	50.6	49.6%	1123	55.4	54.7%	904	46.77	0.4409599	2426	50.6	0.4917881	1,273	48.5	47.7%	940	47.8	45.6%
Yes	2,350	49.4	50.4%	905	44.6	45.3%	1029	53.2	55.9%	2,368	49.4	50.8%	1,351	51.5	52.3%	1,028	52.2	54.4%
**Guardians alcohol/drug issue**																		
No	4443	93.4	93.2%	1880	92.7	92.1%	1,798	93.0	93.0%	4,480	93.5	93.2%	2,404	91.6	91.6%	1,837	93.3	93.4%
Yes	312	6.6	6.8%	148	7.3	7.9%	135	7.0	7.0%	314	6.6	6.8%	220	8.4	8.4%	131	6.7	6.6%
**Self-report childhood health**																		
Better	1696	35.7	36.3%	689	34.0	36.3%	727	37.6	38.6%	1,675	34.9	36.2%	942	35.9	35.9%	738	37.5	37.8%
Same	2,422	50.9	50.3%	1080	53.3	51.6%	979	50.7	49.8%	2,468	51.5	50.4%	1,349	51.4	51.6%	990	50.3	49.5%
Worse	637	13.4	13.5%	259	12.8	12.2%	227	11.7	11.6%	651	13.6	13.3%	333	12.7	12.5%	240	12.2	12.7%
**Self-report childhood finance**																		
Better	380	7.99	8.6%	152	7.5	8.3%	185	9.6	10.2%	383	8.0	9.2%	251	9.6	9.5%	193	9.8	10.2%
Same	2376	50.0	51.6%	1043	51.4	51.4%	1,012	52.4	52.1%	2,406	50.2	51.2%	1,361	51.9	53.1%	1,038	52.7	52.2%
Worse	1,999	42.0	39.8%	833	41.1	40.2%	736	38.1	37.7%	2,005	41.8	39.6%	1,012	38.6	37.4%	737	37.5	37.6%
**Employment Status**																		
Agriculture	1011	21.3	18.6%	348	17.2	15.1%	342	17.7	15.3%	996	20.8	18.0%	387	14.8	13.4%	364	18.5	16.0%
Non-agriculture	1,109	23.3	21.6%	171	8.4	7.5%	728	37.7	38.8%	1,108	23.1	21.2%	1,278	48.7	48.8%	708	36.0	37.0%
Retired or unemployment	2,635	55.4	59.8%	1509	74.4	77.4%	863	44.7	45.9%	2,690	56.1	60.7%	959	36.6	37.8%	896	45.5	47.0%
**Health insurance**																		
No	618	13.0	0.1372776	340	16.8	0.1636157	240	12.4	12.5%	636	13.3	13.8%	328	12.5	12.9%	245	12.5	12.9%
Yes	4137	87.0	86.3%	1688	83.2	83.6%	1693	87.6	87.5%	4158	86.7	86.2%	2,296	87.5	87.1%	1,723	87.6	87.1%
**Income level**																		
Bottom	687	14.5	13.2%	399	19.7	18.1%	178	9.2	8.4%	709	14.8	13.3%	228	8.7	8.3%	195	9.9	8.8%
Middle	1647	34.6	31.2%	706	34.8	31.4%	592	30.6	29.1%	1638	34.2	30.6%	748	28.5	26.9%	593	30.1	29.6%
Top	2,421	50.9	55.6%	923	45.5	50.5%	1163	60.2	62.5%	2,447	51.0	56.0%	1,648	62.8	64.7%	1,180	60.0	61.5%

**Table 3:** Multivariable logistic regression results in the fully adjusted model (model 3)

	2011 Adolescence-Exposed vs. 2018 Childhood-Exposed (*N* = 6,783)				2011 Childhood-Exposed vs. 2018 Fetal-Exposed (*N* = 6,727)				2013 Fetal-Exposed vs. 2018 Non-Exposed (*N* = 4,590)			
	OR	*P*	95%	CI	OR	*P*	95%	CI	OR	*P*	95%	CI
**Age**												
	1.00	0.73	0.98	1.03	1.03	0.04	1.00	1.06	1.03	0.31	0.97	1.10
**GLFF exposure**												
Adolescence-exposed	0.87	0.15	0.72	1.05								
Childhood-exposed	Ref				1.08	0.46	0.88	1.33				
Fetal-exposed					Ref				1.22	0.10	0.96	1.55
Non-exposed									Ref			
**Migration status**												
Static urban residence	1.81	< 0.001	1.42	2.31	1.47	0.04	1.02	2.11	1.33	0.15	0.90	1.96
Rural-to-urban migration	1.26	0.03	1.02	1.56	1.10	0.57	0.79	1.54	1.30	0.09	0.96	1.75
Static rural residence	Ref				Ref				Ref			
Adolescence-exposure X*												
Static urban residence	1.05	0.81	0.72	1.51								
Rural-to-urban migration	1.01	0.94	0.70	1.48								
Static rural residence	Ref											
Childhood-exposure X*												
Static urban residence					1.02	0.91	0.68	1.54				
Rural-to-urban migration					1.05	0.80	0.72	1.54				
Static rural residence					Ref							
Fetal-exposure X*												
Static urban residence									1.06	0.84	0.64	1.75
Rural-to-urban migration									0.81	0.34	0.52	1.25
Static rural residence									Ref			
**Sex**												
Male	Ref				Ref				Ref			
Female	1.06	0.53	0.88	1.28	0.88	0.22	0.73	1.08	0.75	0.04	0.57	0.99
**Education level**												
No formal education	Ref				Ref				Ref			
Below elementary school	1.04	0.71	0.86	1.26	0.95	0.63	0.78	1.17	1.13	0.47	0.81	1.59
Elementary school	1.01	0.92	0.83	1.23	0.88	0.26	0.71	1.10	0.94	0.72	0.68	1.30
Middle school	1.04	0.73	0.83	1.31	1.00	0.97	0.80	1.26	1.11	0.49	0.82	1.51
High school	1.37	0.07	0.98	1.93	0.92	0.57	0.68	1.23	0.78	0.21	0.53	1.15
College and above	1.43	0.08	0.96	2.14	1.43	0.11	0.92	2.22	1.57	0.12	0.90	2.74
**Marital status**												
Unsingle	Ref				Ref				Ref			
Single	0.94	0.46	0.79	1.11	0.79	0.03	0.64	0.97	0.67	0.04	0.46	0.98
**Self-reported health status**												
Good	Ref				Ref				Ref			
Fair	1.99	< 0.001	1.43	2.79	1.84	< 0.001	1.34	2.51	1.99	< 0.001	1.38	2.87
Poor	4.02	< 0.001	2.87	5.64	3.62	< 0.001	2.63	4.98	4.53	< 0.001	3.11	6.61
**Smoke status**												
No	Ref				Ref				Ref			
Yes	0.73	< 0.001	0.61	0.87	0.66	< 0.001	0.55	0.79	0.78	0.06	0.60	1.01
**Alcohol drink status**												
No	Ref				Ref				Ref			
Yes	0.90	0.15	0.77	1.04	0.90	0.19	0.77	1.05	0.84	0.12	0.68	1.04
**Guardians alcohol/drug issue**												
No	Ref				Ref				Ref			
Yes	1.30	0.04	1.01	1.68	1.63	< 0.001	1.27	2.10	1.47	0.02	1.07	2.02
**Self-report childhood health**												
Better	Ref				Ref				Ref			
Same	0.97	0.72	0.84	1.13	0.91	0.20	0.78	1.05	0.82	0.05	0.67	1.00
Worse	1.05	0.63	0.85	1.30	0.83	0.11	0.67	1.04	0.80	0.13	0.59	1.07
**Self-report childhood finance**												
Better	Ref				Ref				Ref			
Same	0.95	0.70	0.74	1.22	0.89	0.34	0.69	1.13	0.75	0.06	0.55	1.02
Worse	1.00	0.99	0.78	1.29	0.94	0.64	0.73	1.21	0.73	0.06	0.54	1.01
**Employment Status**												
Agriculture	Ref				Ref				Ref			
Non-agriculture	0.78	0.04	0.61	0.99	0.83	0.11	0.66	1.04	0.85	0.27	0.63	1.14
Retired or unemployment	1.21	0.05	1.00	1.46	1.24	0.03	1.02	1.51	1.17	0.27	0.89	1.53
**Health insurance**												
No	Ref				Ref				Ref			
Yes	1.19	0.09	0.97	1.45	1.09	0.40	0.89	1.35	1.25	0.13	0.94	1.67
**Income level**												
Bottom	Ref				Ref				Ref			
Middle	1.01	0.94	0.82	1.24	1.00	0.97	0.80	1.26	0.91	0.61	0.64	1.29
Top	1.13	0.22	0.93	1.39	1.18	0.15	0.95	1.47	1.07	0.70	0.77	1.49
**Province Fixed Effect**	Yes				Yes				Yes			

Note: *Intervention term

Model 3: Diabetes_i_ = β_0_+β_1_ GLFE_i_ + β_2_ Migration Status_i_ + β_3_ GLFE_i_ × Migration Status_i_ + β_4_ Covariates_i_ + λ_i_ + ε_i_

**Table 5:** Comparison at different migration statuses

2011 Adolescence-Exposed vs. 2018 Childhood-Exposed	Diff	95%	CI
Static urban residence	-0.016	-0.071	0.038
Rural-to-urban migration	-0.018	-0.063	0.028
Static rural residence	-0.017	-0.040	0.006
**2011 Childhood-Exposed vs. 2018 Fetal-Exposed**	**Diff**	**95%**	**CI**
Static urban residence	0.015	-0.038	0.068
Rural-to-urban migration	0.016	-0.025	0.058
Static rural residence	0.009	-0.015	0.033
**2013 Fetal-Exposed vs. 2018 Non-Exposed**	**Diff**	**95%**	**CI**
Static urban residence	0.031	-0.024	0.087
Rural-to-urban migration	-0.002	-0.044	0.041
Static rural residence	0.020	-0.004	0.044

### Demographic characteristics

Table 2 describes the variation in socioeconomic and behavioral characteristics in three different age groups. Based on the weighted prevalence rate, in the first age sample (Group 1), the prevalence of diabetes is 17.5% and 17.1% among 2018 childhood-exposed individuals and 2011 adolescence-exposed individuals, respectively. In the second age sample (Group 2), the prevalence of diabetes is 15.2% and 16.7% among 2011 childhood-exposed individuals and 2018 fetal-exposed individuals, respectively. In the third sample (Group 3), the prevalence of diabetes is 11.4% and 14.5% among 2013 fetal-exposed individuals and 2018 non-exposed individuals, respectively. In each age group, the urban/rural/migration statuses between the two exposure windows are similar.

### GLFF exposure effects

For each age stratum, we compared the adult-onset diabetes risk between the adjacent GLFF exposure windows using three multivariable logistic regression models, as detailed in formulas Model 1-Moddel 3, and the results are presented in Supplement Table 3-Supplement Table 6. In summary, Group 1 showed non-significant differences in adult-onset diabetes risk between adolescence and childhood exposure across Model 1-Model 3. Similarly, Group 2 showed non-significant differences in adult-onset diabetes risk between childhood and fetal exposure across Model 1-Model 3. Also, Group 3 revealed marginal insignificant differences in adult-onset diabetes risk between fetal- and non-exposure in Model 2 and Model 3.

Due to the extremely small proportions of urban-to-rural migrants within each age stratum, we further excluded the rural-to-urban migrants in the fully adjusted multivariable logistical regression. The exclusion of urban-to-rural migrants did not change the results. As shown in Table 3, the window of GLFF exposure was not associated with adult-onset diabetes risk across the three age-stratum, after adjusting for provinces, demographics, health statuses, and childhood growth environment.

### Urban/rural/migration effects

As shown in Table 3, the analyses of Group 1 and Group 2 supported the effects of urban/rural/migration statuses on adult-onset diabetes in the fully adjusted model (Model 3). Specifically, among those aged between 61 and 72 (Group 1), static urban residence had a 1.81-fold increase in the odds of adult-onset diabetes compared to those with static rural residence (Odds Ratio[OR] = 1.81, *p* < 0.001, 95%CI=[1.42, 2.31]). Within the same age group (Group 1), rural-to-urban migrants had a 1.26-fold increase in the odds of adult-onset diabetes, compared to the static rural residents (OR = 1.26, *p* = 0.03, 95CI=[1.02, 1.56]). Among those aged between 54 and 64 (Group 2), static urban residence, again, had a 1.47-fold increase in the odds of adult-onset diabetes, compared to those with static rural residence (OR = 1.47, *p* < 0.04, 95%CI=[1.02, 2.11]); whereas, the effects of rural-to-urban migration on adults-onset diabetes were not significant in this age group (Group 2). Finally, among those aged between 50 and 56 years (Group 3), the effects of urban/rural/migration statuses on the risk of adult-onset diabetes were not observed. The synergistic urban environmental effect on diabetes risk was more prominent as adults get older (Group 1).

### Lack of interaction between GLFF exposure and urban/rural/migration statuses

As described in Table 3, the interaction terms of GLFF exposure and urban/rural/migration statuses were not significant across all three age-stratification groups. Table 4 describes the convolution of GLFF exposure and urban/rural/migration statuses on current prevalence of diabetes in China. In Group 1, static urban residents had a heightened risk of adult-onset diabetes than their rural counterparts, regardless of childhood-exposure or adolescence-exposure to GLFF. In Group 2, childhood-exposed individuals with static urban residence demonstrated a higher risk of diabetes than childhood-exposed individuals with static rural residence. However, among those aged between 50 and 56 years (Group 3), the effects of urban/rural/migration statuses on the risk of adult-onset diabetes were not observed on both fetal-exposed and non-exposed individuals. As shown in Table 5, the margin estimation did not support the interaction effects between GLFF exposure and urban/rural/migration statuses across all three age-stratification groups.

**Table 4:** Comparison between different migration statuses

2011 Adolescence-Exposed vs. 2018 Childhood-Exposed	Diff	95%	CI
**2018 Childhood-Exposed**			
Static rural residence (Ref.)			
Static urban residence	0.089	0.049	0.128
Rural-to-urban migration	0.032	0.002	0.061
**2011 Adolescence-Exposed**			
Static rural residence (Ref.)			
Static urban residence	0.090	0.039	0.140
Rural-to-urban migration	0.031	-0.011	0.073
**2011 Childhood-Exposed vs. 2018 Fetal-Exposed**	**Diff**	**95%**	**CI**
**2018 Fetal-Exposed**			
Static rural residence (Ref.)			
Static urban residence	0.050	-0.001	0.101
Rural-to-urban migration	0.012	-0.029	0.052
**2011 Childhood-Exposed**			
Static rural residence (Ref.)			
Static urban residence	0.056	0.020	0.092
Rural-to-urban migration	0.019	-0.009	0.046
**2013 Fetal-Exposed vs. 2018 Non-Exposed**	**Diff**	**95%**	**CI**
**2018 Non-Exposed**			
Static rural residence (Ref.)			
Static urban residence	0.030	-0.014	0.073
Rural-to-urban migration	0.027	-0.006	0.059
**2011 Fetal-Exposed**			
Static rural residence (Ref.)			
Static urban residence	0.041	-0.008	0.090
Rural-to-urban migration	0.005	-0.033	0.043

### Sensitivity analysis

Without the adjustment of childhood growth environment, fetal exposure to GLFF was associated with an increased risk of adult-onset diabetes (OR = 1.261, 95% CI = [1.007, 1.579]), when controlling for the provinces, demographics, and health statuses. Yet, adding urban-rural migration statuses into the model diminished the GLFF effects on diabetes risk, with no observed interaction effect (Supplement Table 7). The conditional analysis revealed that the urban effect on diabetes risk outweighs the impact of GLFF exposure on diabetes risk. In particular, among individuals with fetal GLFF exposure, those with static urban status exhibited a higher diabetes risk compared to their rural counterparts (Supplement Table 8). Yet, when conditioning on urban-rural statuses, the GLFF exposure effects on diabetes risk were not observed anymore (Supplement Table 9).

## Discussion

### Early life exposure to GLFF

Cumulative studies surrounding the investigation of GLFF exposure on diabetes risk indicated that those who were exposed to GLFF during fetal status had a higher risk of adult-onset diabetes, compared to those who were never exposed to GLFF [14, 29]. The constant debate regarding whether GLFF exposure windows play a critical role in diabetes risk was carefully examined in the current study. We addressed three major critics surrounding this debate: exposure window, age, and geographic confounding effects. After controlling for the provinces, demographics, health statuses and the proxy of childhood growth environments, fetal exposure to GLFF was not associated with adult-onset diabetes risk (*p* = 0.10), compared to those who never exposed to GLFF. Additionally, we also examined other early life exposure to GLFF, and found that adolescence exposure and childhood exposure to GLFF was not associated with adult-onset diabetes risk, compared to those who are childhood-exposed and fetal-exposed, respectively.

The sensitivity analysis suggests that fetal exposure to GLFF might have a traceable effect on adult-onset diabetes risk. Yet, the growth environment and urban lifestyle could outweigh or further confound the impact of GLFF exposure on adult-onset diabetes risk. Specifically, across the three age-stratification groups, guardians’ alcohol/drug issues were significantly associated with the risk of adult-onset diabetes, whereas the impact of GLFF exposure on adult-onset diabetes risk was unobservable in the fully adjusted model. Though, it is premature to conclude this association because there is a lack of context showing the cause, length, and intensity of parental alcohol/drug issues and the degree to which parental alcohol/drug issues impact children’s growth environments. Still, our findings support Bronfenbrenner’s Socio-Ecological Theory, which elucidates the importance and the complexity of the early-life growth environment in the continuum of disease development [30, 31]. Future studies with careful design of mediation analysis and measures of contextual variables are warranted to understand the underlying mechanisms.

### Urban effects on diabetes prevalence in China

Our study underscores the significant impact of static urban residence and rural-to-urban migration on the current prevalence of diabetes in China. We observed that individuals residing permanently in urban settings demonstrated a higher risk of diabetes across all three age-stratified groups, in contrast to their counterparts in static rural environments. Even though urban areas were reportedly less affected by the GLFF, we found that static urban residents experienced a higher risk of adult-onset diabetes than static rural residents. This contrast could be attributed to distinct lifestyle factors, dietary habits, stress levels, and mental health issues prevalent in metropolitan areas. Research indicates that urban dwellers engaged in more sedentary activities and less physical activities compared to those in rural areas [32]. Moreover, the contemporary urban environment and industrialization lead to a shift in dietary patterns, moving away from a traditional, whole-food diets, that are rich in vegetables, grains, and fibers, to more processed foods that are high in refined sugar and saturated fats but lower in nutritional value [33]. In addition, urban living conditions, characterized by job-related pressures, traffic congestion, noise, and overcrowding, contribute to elevated stress levels, which are known to increase the risk of developing diabetes and metabolic syndrome [34, 35].

The Thrifty Phenotype Hypothesis theorizes that the thrifty phenotype (i.e., efficiency at storing energy as fat) adapted during fetal famine exposure as an advantage becomes detrimental in the modern food-abundance environment [36, 37]. Although our findings did not fully support the Thrifty Phenotype Hypothesis due to the lack of interaction (URBAN*GLFF) effect, our findings of traceable effects of fetal GLFF exposure and strong impacts of urban-rural statuses (Supplement Table 7) could not reject the Thrifty Phenotype Hypothesis either. The underlying mechanisms of Thrifty Phenotype Hypothesis warrant future research to be confirmed. Moreover, our finding does not fully support Li and Lumey’s recent meta-analysis, where they concluded that the effect of GLFF exposures on diabetes risk did not differ between rural and urban residents [22]. In fact, as demonstrated in Table 4, our finding is coherent with Wang and colleagues’ conclusion that those who grew up in the urban areas (defined by the gross domestic product per capita) had even higher risk of diabetes compared to those who grew up in the rural areas [14]. Furthermore, consistent with prior studies, our results further confirm that the prevalence of diabetes is higher in urban areas than rural areas in China [19, 38].

Drawing from Bronfenbrenner’s Socio-Ecological Theory, chronic illnesses like diabetes have multifaceted origins, shaped by immediate environments (urban/rural/migration statuses and childhood growth environment) and broader cultural and historical contexts (GLFF exposure). While all layers of the social-ecological systems play roles in the continuum of metabolic dysregulation in the aging process, our findings highlight an important notion that the innate systems might have more salient effects than the outer systems, although the outer systems have a broader impact on the population than the innate systems.

### Policy considerations

Urbanization, while offering better job opportunities and economic growth, brings challenges like processed and refined food, overcrowding, and lifestyle changes. Our findings provide several policy implications. First, prenatal nutrition is critical for fetal metabolic development, where providing adequate food might be insufficient; instead, ensuring a high-quality food environment and healthy food supply is a crucial step to addressing perinatal nutrition. Second, public health initiatives must ensure food standards remain evidence based. For instance, the recent proposed changes to China’s nutrition labels emphasize the inclusion of sugar and saturated fat details [39]. Finally, current labeling standards don’t cover street food or restaurant-packaged meals. Thus, smart food choices, health education, and preventive care services should be implemented in conjunction with food labeling policies.

### Limitations

Our study acknowledges several limitations. First, we did not include critical diabetes risk factors as covariates, such as genetic variants (e.g., polygenic risk scores, PGS) and dietary habits. This omission could potentially influence the results. Second, due to the structure of the CHARLS survey waves, our analysis was confined to comparing adjacent birth cohorts as detailed in Table 1. This restriction hindered our ability to directly compare groups like the adolescent-exposed and childhood-exposed individuals with the non-exposed group, which might have yielded more comprehensive insights. Nonetheless, this limitation offers an opportunity for future studies, especially as subsequent CHARLS survey waves become available. Third, each CHARLS survey wave reflects its unique socioeconomic contexts that could affect the awareness, knowledge, and diagnosis of diabetes, which could synergistically confound the prevalence of diabetes. In addition, our categorization of urban-rural migration status was based on hukou records and the residential places when the survey occurred, without contextual information pertaining to the actual length of rural and urban residency. These unmeasurable factors remain beyond our control, thus introducing potential biases. Fourth, the small proportions of urban-to-rural migrants within each age stratum yield limited statistical powers. Hence, caution should be taken when interpreting the generalizability of findings in Table 3, as we precluded the rural-to-urban migrants in the fully adjusted multivariable logistical regression. The results of the full sample that includes urban-to-rural migrants are presented in Supplement Table 6. Furthermore, our primary outcome, diabetes, was based on the diagnosis of diabetes collected from participants, where underdiagnosis bias might occur. Nevertheless, the prevalence of diabetes in our study is consistent with the existing literature of diabetes prevalence in China [40–42]. Lastly, our approach of using birth cohorts to identify early-life famine exposure, adjusted for provincial and urban-rural disparities, may not fully consider individuals who, due to familial wealth or privilege, had access to adequate nutrition during the Great Leap Forward Famine and were thus not exposed to its effects. This factor could skew our understanding of the famine’s impact on diabetes risk.

## Conclusion

Fetal famine exposure to GLFF predisposes individuals to a greater risk of diabetes, however, this effect might be confounded by the geographic dynamics in rural and urban statuses and childhood growth environments. Although static urban residence was associated with an increased risk of diabetes in later life, interaction effects between GLFF exposure and urban/rural/migration statuses were not supported in current study.

### Electronic supplementary material

Below is the link to the electronic supplementary material.

Supplementary Material 1

## Data Availability

Program code supporting the findings of this study are available from the author on request. All users who analyze CHARLS data should follow the China Health and Retirement Longitudinal Study Data Access User Agreement. All data can be accessed through http://charls.pku.edu.cn/.
