# Supplementary material for: Great leap forward famine exposure and urban-rural migration convolute the modern prevalence of diabetes in China
Source: J Health Popul Nutr. 2024 Jul 30;43:109. doi: 10.1186/s41043-024-00596-2 (PMC11290054; doi:10.1186/s41043-024-00596-2)
Supplement: Supplementary file 1 — Supplementary Material 1 [file 41043_2024_596_MOESM1_ESM.docx]

**Supplement Table 1: Distribution of missing values**

| **Variable** | **No. Missing** | **No. Non-missing** | **values** | **Min** | **Max** |
| --- | --- | --- | --- | --- | --- |
| Urbanicity | 1,698 | 22,989 | 4 | 0 | 3 |
| Sex | 2 | 24,685 | 2 | 1 | 2 |
| Education level | 10 | 24,677 | 6 | 1 | 6 |
| Marital Status | 3 | 24,684 | 2 | 1 | 2 |
| Self-reported health status | 779 | 23,908 | 3 | 1 | 3 |
| Smoke Status | 151 | 24,536 | 2 | 0 | 1 |
| Alcohol drink Status | 127 | 24,560 | 2 | 0 | 1 |
| Guardians alcohol/drug issue | 5,573 | 19,114 | 2 | 0 | 1 |
| Self-report childhood health | 5,200 | 19,495 | 3 | 0 | 2 |
| Self-report childhood finance | 5,192 | 19,495 | 3 | 0 | 2 |
| Employment status | 122 | 24,565 | 3 | 1 | 3 |
| Health insurance | 247 | 24,440 | 2 | 0 | 1 |
| Income level | 1,380 | 23,307 | 3 | 1 | 3 |

**Supplement Table 2: Patterns of missing data.**

|  | **2011 Adolescence-exposed vs. 2018 Childhood-exposed** | | | **2011 Childhood-exposed vs. 2018 Fetal-exposed** | | | **2013 Fetal-exposed vs. 2018 Non-exposed** | | |
| --- | --- | --- | --- | --- | --- | --- | --- | --- | --- |
|  | **Missing value status** | | | **Missing value status** | | | **Missing value status** | | |
|  | **No** | **Yes** | **P-value** | **No** | **Yes** | **P-value** | **No** | **Yes** | **P-value** |
| **Diabetes status** | **Urban-Rural Status** | | | **Urban-Rural Status** | | | **Urban-Rural Status** | | |
| No | 7,499 | 103 | 0.367 | 7,578 | 74 | 0.46 | 5,400 | 221 | 0.076 |
| Yes | 1,551 | 26 |  | 1,431 | 11 |  | 802 | 22 |  |
|  | **Self-reported health status** | | | **Self-reported health status** | | | **Self-reported health status** | | |
| No | 7,509 | 93 | 0.116 | 7,529 | 123 | 0.469 | 5,564 | 57 | 0.908 |
| Yes | 1,565 | 12 |  | 1,415 | 27 |  | 816 | 8 |  |
|  | **Smoke status** | | | **Smoke status** | | | **Smoke status** | | |
| No | 7,586 | 16 | 0.216 | 7,632 | 20 | 0.57 | 5,618 | 3 | 0.068 |
| Yes | 1,576 | 1 |  | 1,437 | 5 |  | 822 | 2 |  |
|  | **Alcohol drink Status** | | | **Drink status** | | | **Drink status** | | |
| No | 7,590 | 12 | 0.114 | 7,626 | 26 | 0.083 | 5,618 | 3 | 0.464 |
| Yes | 1,577 | 0 |  | 1,441 | 1 |  | 823 | 1 |  |
|  | **Guardians alcohol/drug issue** | |  | **Guardians alcohol/drug issue** | |  | **Guardians alcohol/drug issue** | |  |
| No | 6,527 | 1,075 | 0.073 | 6,410 | 1,242 | 0.017 | 4,762 | 859 | 0.472 |
| Yes | 1,381 | 196 |  | 1,244 | 198 |  | 706 | 118 |  |
|  | **Self-report childhood health** | |  | **Self-report childhood health** | |  | **Self-report childhood health** | |  |
| No | 6,623 | 979 | 0.061 | 6,474 | 1,178 | 0.007 | 4,799 | 822 | 0.488 |
| Yes | 1,401 | 176 |  | 1,260 | 182 |  | 711 | 113 |  |
|  | **Self-report childhood finance** | |  | **Self-report childhood finance** | |  | **Self-report childhood finance** | |  |
| No | 6,630 | 972 | 0.088 | 6,485 | 1,167 | 0.021 | 4,796 | 825 | 0.464 |
| Yes | 1,400 | 177 |  | 1,256 | 186 |  | 711 | 113 |  |
|  | **Employment status** | | | **Employment status** | | | **Employment status** | | |
| No | 7,595 | 7 | 0.726 | 7,639 | 13 | 0.372 | 5,617 | 4 | 0.629 |
| Yes | 1,576 | 1 |  | 1,441 | 1 |  | 823 | 1 |  |
|  | **Health insurance** | | | **Health insurance** | | | **Health insurance** | | |
| No | 7,597 | 5 | 0.973 | 7,650 | 2 | 0.539 | 5,615 | 6 | 0.301 |
| Yes | 1,576 | 1 |  | 1,442 | 0 |  | 822 | 2 |  |
|  | **Income level** | | | **Income level** | | | **Income level** | | |
| No | 7,383 | 219 | 0.738 | 7,442 | 210 | 0.039 | 5,477 | 144 | 0.428 |
| Yes | 1,534 | 43 |  | 1,416 | 26 |  | 799 | 25 |  |

**Supplement Table 3: Multivariable logistic regression of Group 1 (2011 adolescence-exposed vs. 2018 childhood-exposed) in Model 1 and Model 2 with all four urban-rural statuses.**

|  | **2011 Adolescence-Exposed vs. 2018 Childhood-Exposed** | | | | | | | |
| --- | --- | --- | --- | --- | --- | --- | --- | --- |
|  | **Model 1** |  |  |  | **Model 2** |  |  |  |
| **N=6,783** | **OR** | **P** | **95%** | **CI** | **OR** | **P** | **95%** | **CI** |
| **Age** |  |  |  |  |  |  |  |  |
|  | 1.01 | 0.61 | 0.98 | 1.03 | 1.00 | 0.77 | 0.98 | 1.03 |
| **GLFF exposure** |  |  |  |  |  |  |  |  |
| Adolescence-exposed | 0.88 | 0.10 | 0.76 | 1.03 | 0.88 | 0.09 | 0.75 | 1.02 |
| Childhood-exposed | Ref |  |  |  | Ref |  |  |  |
| Fetal-exposed |  |  |  |  |  |  |  |  |
| Non-exposed |  |  |  |  |  |  |  |  |
| **Urban/Rural/migration Status** |  |  |  |  |  |  |  |  |
| Static urban residence |  |  |  |  | 1.86 | 0.00 | 1.51 | 2.30 |
| Rural-to-urban migration |  |  |  |  | 1.27 | 0.01 | 1.06 | 1.52 |
| Static rural residence |  |  |  |  | Ref |  |  |  |
| **Sex** |  |  |  |  |  |  |  |  |
| Male | Ref |  |  |  | Ref |  |  |  |
| Female | 1.09 | 0.37 | 0.91 | 1.30 | 1.07 | 0.46 | 0.89 | 1.29 |
| **Education level** |  |  |  |  |  |  |  |  |
| No formal education | Ref |  |  |  | Ref |  |  |  |
| Below elementary school | 1.07 | 0.46 | 0.89 | 1.30 | 1.03 | 0.76 | 0.85 | 1.25 |
| Elementary school | 1.09 | 0.38 | 0.90 | 1.32 | 1.01 | 0.94 | 0.83 | 1.23 |
| Middle school | 1.22 | 0.07 | 0.98 | 1.53 | 1.04 | 0.75 | 0.83 | 1.30 |
| High school | 1.63 | 0.00 | 1.18 | 2.26 | 1.32 | 0.11 | 0.94 | 1.84 |
| College and above | 1.94 | 0.00 | 1.35 | 2.77 | 1.40 | 0.08 | 0.96 | 2.04 |
| **Marital status** |  |  |  |  |  |  |  |  |
| Unsingle | Ref |  |  |  | Ref |  |  |  |
| Single | 0.94 | 0.45 | 0.79 | 1.11 | 0.95 | 0.57 | 0.80 | 1.13 |
| **Self-reported health status** |  |  |  |  |  |  |  |  |
| Good | Ref |  |  |  | Ref |  |  |  |
| Fair | 1.95 | 0.00 | 1.41 | 2.71 | 1.98 | 0.00 | 1.43 | 2.75 |
| Poor | 3.71 | 0.00 | 2.67 | 5.15 | 3.94 | 0.00 | 2.83 | 5.49 |
| **Smoke status** |  |  |  |  |  |  |  |  |
| No | Ref |  |  |  | Ref |  |  |  |
| Yes | 0.72 | 0.00 | 0.60 | 0.85 | 0.74 | 0.00 | 0.62 | 0.87 |
| **Alcohol drink status** |  |  |  |  |  |  |  |  |
| No | Ref |  |  |  | Ref |  |  |  |
| Yes | 0.87 | 0.07 | 0.75 | 1.01 | 0.87 | 0.07 | 0.75 | 1.01 |
| **Guardians alcohol/drug issue** |  |  |  |  |  |  |  |  |
| No | Ref |  |  |  | Ref |  |  |  |
| Yes | 1.29 | 0.05 | 1.00 | 1.65 | 1.29 | 0.05 | 1.00 | 1.65 |
| **Self-report childhood health** |  |  |  |  |  |  |  |  |
| Better | Ref |  |  |  | Ref |  |  |  |
| Same | 0.98 | 0.80 | 0.85 | 1.13 | 0.98 | 0.79 | 0.85 | 1.13 |
| Worse | 1.08 | 0.48 | 0.88 | 1.32 | 1.07 | 0.52 | 0.87 | 1.31 |
| **Self-report childhood finance** |  |  |  |  |  |  |  |  |
| Better | Ref |  |  |  | Ref |  |  |  |
| Same | 0.90 | 0.41 | 0.71 | 1.15 | 0.94 | 0.64 | 0.74 | 1.20 |
| Worse | 0.95 | 0.68 | 0.74 | 1.22 | 1.00 | 0.99 | 0.78 | 1.28 |
| **Employment Status** |  |  |  |  |  |  |  |  |
| Agriculture | Ref |  |  |  | Ref |  |  |  |
| Non-agriculture | 0.81 | 0.08 | 0.63 | 1.03 | 0.79 | 0.06 | 0.62 | 1.01 |
| Retired or unemployment | 1.33 | 0.00 | 1.11 | 1.60 | 1.20 | 0.05 | 1.00 | 1.45 |
| **Health insurance** |  |  |  |  |  |  |  |  |
| No | Ref |  |  |  | Ref |  |  |  |
| Yes | 1.17 | 0.11 | 0.96 | 1.42 | 1.21 | 0.05 | 1.00 | 1.47 |
| **Income level** |  |  |  |  |  |  |  |  |
| Bottom | Ref |  |  |  | Ref |  |  |  |
| Middle | 1.04 | 0.68 | 0.85 | 1.28 | 1.01 | 0.90 | 0.83 | 1.24 |
| Top | 1.23 | 0.04 | 1.01 | 1.50 | 1.12 | 0.26 | 0.92 | 1.37 |
| **Province Fixed Effect** | Yes |  |  |  | Yes |  |  |  |

Note:

Model 1: ${Diabetes}_{i}=\beta_{0}+\beta_{1}{GLFE}_{i}+\beta_{2}{Covariates}_{i}+\lambda_{i}+\varepsilon_{i}\left( 1 \right)$

Model 2: ${Diabetes}_{i}=\beta_{0}+\beta_{1}{GLFE}_{i}+\beta_{2}{Migration Status}_{i}+\beta_{3}{Covariates}_{i}+\lambda_{i}+\varepsilon_{i}(2)$

**Supplement Table 4: Multivariable logistic regression of Group 2 (2011 childhood-exposed vs. 2018 fetal-exposed)** **in Model 1 and Model 2 with all four urban-rural statuses.**

|  | **2011 Childhood-Exposed vs. 2018 Fetal-Exposed** | | | | | | | |
| --- | --- | --- | --- | --- | --- | --- | --- | --- |
|  | **Model 1** |  |  |  | **Model 2** |  |  |  |
| **N=6,727** | **OR** | **P** | **95%** | **CI** | **OR** | **P** | **95%** | **CI** |
| **Age** |  |  |  |  |  |  |  |  |
|  | 1.03 | 0.04 | 1.00 | 1.06 | 1.03 | 0.06 | 1.00 | 1.05 |
| **GLFF exposure** |  |  |  |  |  |  |  |  |
| Adolescence-exposed |  |  |  |  |  |  |  |  |
| Childhood-exposed | 1.11 | 0.19 | 0.95 | 1.31 | 1.09 | 0.29 | 0.93 | 1.28 |
| Fetal-exposed | Ref |  |  |  | Ref |  |  |  |
| Non-exposed |  |  |  |  |  |  |  |  |
| **Urban/Rural/migration Status** |  |  |  |  |  |  |  |  |
| Static urban residence |  |  |  |  | 1.51 | 0.00 | 1.22 | 1.88 |
| Rural-to-urban migration |  |  |  |  | 1.15 | 0.15 | 0.95 | 1.38 |
| Static rural residence |  |  |  |  | Ref |  |  |  |
| **Sex** |  |  |  |  |  |  |  |  |
| Male | Ref |  |  |  | Ref |  |  |  |
| Female | 0.90 | 0.30 | 0.74 | 1.10 | 0.89 | 0.27 | 0.74 | 1.09 |
| **Education level** |  |  |  |  |  |  |  |  |
| No formal education | Ref |  |  |  | Ref |  |  |  |
| Below elementary school | 0.98 | 0.81 | 0.80 | 1.19 | 0.95 | 0.61 | 0.77 | 1.16 |
| Elementary school | 0.92 | 0.45 | 0.74 | 1.14 | 0.88 | 0.25 | 0.71 | 1.09 |
| Middle school | 1.08 | 0.50 | 0.87 | 1.34 | 0.98 | 0.85 | 0.78 | 1.23 |
| High school | 1.02 | 0.90 | 0.77 | 1.36 | 0.89 | 0.46 | 0.67 | 1.20 |
| College and above | 1.78 | 0.01 | 1.19 | 2.64 | 1.43 | 0.09 | 0.94 | 2.17 |
| **Marital status** |  |  |  |  |  |  |  |  |
| Unsingle | Ref |  |  |  | Ref |  |  |  |
| Single | 0.79 | 0.03 | 0.64 | 0.98 | 0.80 | 0.03 | 0.65 | 0.98 |
| **Self-reported health status** |  |  |  |  |  |  |  |  |
| Good | Ref |  |  |  | Ref |  |  |  |
| Fair | 1.88 | 0.00 | 1.38 | 2.56 | 1.89 | 0.00 | 1.38 | 2.57 |
| Poor | 3.59 | 0.00 | 2.62 | 4.91 | 3.70 | 0.00 | 2.70 | 5.07 |
| **Smoke status** |  |  |  |  |  |  |  |  |
| No | Ref |  |  |  | Ref |  |  |  |
| Yes | 0.65 | 0.00 | 0.54 | 0.78 | 0.66 | 0.00 | 0.55 | 0.79 |
| **Alcohol drink status** |  |  |  |  |  |  |  |  |
| No | Ref |  |  |  | Ref |  |  |  |
| Yes | 0.89 | 0.13 | 0.76 | 1.03 | 0.89 | 0.12 | 0.76 | 1.03 |
| **Guardians alcohol/drug issue** |  |  |  |  |  |  |  |  |
| No | Ref |  |  |  | Ref |  |  |  |
| Yes | 1.62 | 0.00 | 1.26 | 2.07 | 1.62 | 0.00 | 1.27 | 2.08 |
| **Self-report childhood health** |  |  |  |  |  |  |  |  |
| Better | Ref |  |  |  | Ref |  |  |  |
| Same | 0.91 | 0.22 | 0.79 | 1.06 | 0.91 | 0.21 | 0.79 | 1.06 |
| Worse | 0.84 | 0.13 | 0.68 | 1.05 | 0.84 | 0.11 | 0.67 | 1.04 |
| **Self-report childhood finance** |  |  |  |  |  |  |  |  |
| Better | Ref |  |  |  | Ref |  |  |  |
| Same | 0.85 | 0.18 | 0.66 | 1.08 | 0.88 | 0.28 | 0.69 | 1.12 |
| Worse | 0.91 | 0.45 | 0.71 | 1.16 | 0.94 | 0.64 | 0.73 | 1.21 |
| **Employment Status** |  |  |  |  |  |  |  |  |
| Agriculture | Ref |  |  |  | Ref |  |  |  |
| Non-agriculture | 0.85 | 0.16 | 0.67 | 1.07 | 0.83 | 0.12 | 0.66 | 1.05 |
| Retired or unemployment | 1.33 | 0.00 | 1.10 | 1.60 | 1.24 | 0.03 | 1.02 | 1.50 |
| **Health insurance** |  |  |  |  |  |  |  |  |
| No | Ref |  |  |  | Ref |  |  |  |
| Yes | 1.09 | 0.40 | 0.89 | 1.34 | 1.11 | 0.32 | 0.90 | 1.36 |
| **Income level** |  |  |  |  |  |  |  |  |
| Bottom | Ref |  |  |  | Ref |  |  |  |
| Middle | 1.02 | 0.84 | 0.82 | 1.28 | 1.01 | 0.96 | 0.80 | 1.26 |
| Top | 1.24 | 0.06 | 1.00 | 1.53 | 1.17 | 0.16 | 0.94 | 1.46 |
| **Province Fixed Effect** | Yes |  |  |  | Yes |  |  |  |

Note:

Model 1: ${Diabetes}_{i}=\beta_{0}+\beta_{1}{GLFE}_{i}+\beta_{2}{Covariates}_{i}+\lambda_{i}+\varepsilon_{i}\left( 1 \right)$

Model 2: ${Diabetes}_{i}=\beta_{0}+\beta_{1}{GLFE}_{i}+\beta_{2}{Migration Status}_{i}+\beta_{3}{Covariates}_{i}+\lambda_{i}+\varepsilon_{i}(2)$

**Supplement Table 5: Multivariable logistic regression of Group 3 (2013 fetal-exposed vs. 2018 non-exposed) in Model 1 and Model 2 with all four urban-rural statuses.**

|  | **2013 Fetal-Exposed vs. 2018 Non-Exposed** | | | | | | | |
| --- | --- | --- | --- | --- | --- | --- | --- | --- |
|  | **Model 1** |  |  |  | **Model 2** |  |  |  |
|  | **OR** | **P** | **95%** | **CI** | **OR** | **P** | **95%** | **CI** |
| **N=4,590** |  |  |  |  |  |  |  |  |
| **Age** |  |  |  |  |  |  |  |  |
|  | 1.03 | 0.29 | 0.97 | 1.10 | 1.03 | 0.30 | 0.97 | 1.10 |
| **GLFF exposure** |  |  |  |  |  |  |  |  |
| Adolescence-exposed |  |  |  |  |  |  |  |  |
| Childhood-exposed |  |  |  |  |  |  |  |  |
| Fetal-exposed | 1.18 | 0.08 | 0.98 | 1.42 | 1.17 | 0.09 | 0.98 | 1.41 |
| Non-exposed | Ref |  |  |  | Ref |  |  |  |
| **Urban/Rural/migration Status** |  |  |  |  |  |  |  |  |
| Static urban residence |  |  |  |  | 1.42 | 0.02 | 1.06 | 1.89 |
| Rural-to-urban migration |  |  |  |  | 1.18 | 0.17 | 0.93 | 1.50 |
| Static rural residence |  |  |  |  | Ref |  |  |  |
| **Sex** |  |  |  |  |  |  |  |  |
| Male | Ref |  |  |  | Ref |  |  |  |
| Female | 0.79 | 0.08 | 0.60 | 1.03 | 0.78 | 0.07 | 0.59 | 1.02 |
| **Education level** |  |  |  |  |  |  |  |  |
| No formal education | Ref |  |  |  | Ref |  |  |  |
| Below elementary school | 1.15 | 0.42 | 0.82 | 1.60 | 1.13 | 0.46 | 0.81 | 1.58 |
| Elementary school | 0.98 | 0.90 | 0.71 | 1.35 | 0.96 | 0.79 | 0.70 | 1.32 |
| Middle school | 1.16 | 0.33 | 0.86 | 1.56 | 1.10 | 0.52 | 0.82 | 1.49 |
| High school | 0.83 | 0.33 | 0.57 | 1.21 | 0.75 | 0.14 | 0.51 | 1.10 |
| College and above | 1.67 | 0.05 | 1.00 | 2.80 | 1.33 | 0.31 | 0.77 | 2.30 |
| **Marital status** |  |  |  |  |  |  |  |  |
| Unsingle | Ref |  |  |  | Ref |  |  |  |
| Single | 0.68 | 0.04 | 0.47 | 0.98 | 0.66 | 0.03 | 0.45 | 0.96 |
| **Self-reported health status** |  |  |  |  |  |  |  |  |
| Good | Ref |  |  |  | Ref |  |  |  |
| Fair | 2.06 | 0.00 | 1.43 | 2.97 | 2.09 | 0.00 | 1.45 | 3.01 |
| Poor | 4.56 | 0.00 | 3.13 | 6.64 | 4.70 | 0.00 | 3.22 | 6.85 |
| **Smoke status** |  |  |  |  |  |  |  |  |
| No | Ref |  |  |  | Ref |  |  |  |
| Yes | 0.79 | 0.07 | 0.61 | 1.02 | 0.80 | 0.08 | 0.62 | 1.03 |
| **Alcohol drink status** |  |  |  |  |  |  |  |  |
| No | Ref |  |  |  | Ref |  |  |  |
| Yes | 0.84 | 0.12 | 0.68 | 1.04 | 0.84 | 0.12 | 0.68 | 1.04 |
| **Guardians alcohol/drug issue** |  |  |  |  |  |  |  |  |
| No | Ref |  |  |  | Ref |  |  |  |
| Yes | 1.46 | 0.02 | 1.07 | 2.00 | 1.48 | 0.02 | 1.08 | 2.03 |
| **Self-report childhood health** |  |  |  |  |  |  |  |  |
| Better | Ref |  |  |  | Ref |  |  |  |
| Same | 0.81 | 0.03 | 0.66 | 0.98 | 0.81 | 0.04 | 0.67 | 0.99 |
| Worse | 0.79 | 0.11 | 0.59 | 1.05 | 0.79 | 0.11 | 0.59 | 1.05 |
| **Self-report childhood finance** |  |  |  |  |  |  |  |  |
| Better | Ref |  |  |  | Ref |  |  |  |
| Same | 0.73 | 0.04 | 0.54 | 0.98 | 0.74 | 0.05 | 0.55 | 1.01 |
| Worse | 0.72 | 0.04 | 0.52 | 0.98 | 0.73 | 0.05 | 0.53 | 1.00 |
| **Employment Status** |  |  |  |  |  |  |  |  |
| Agriculture | Ref |  |  |  | Ref |  |  |  |
| Non-agriculture | 0.91 | 0.51 | 0.68 | 1.21 | 0.87 | 0.34 | 0.65 | 1.16 |
| Retired or unemployment | 1.25 | 0.11 | 0.95 | 1.63 | 1.17 | 0.26 | 0.89 | 1.54 |
| **Health insurance** |  |  |  |  |  |  |  |  |
| No | Ref |  |  |  | Ref |  |  |  |
| Yes | 1.22 | 0.18 | 0.92 | 1.62 | 1.24 | 0.14 | 0.93 | 1.65 |
| **Income level** |  |  |  |  |  |  |  |  |
| Bottom | Ref |  |  |  | Ref |  |  |  |
| Middle | 0.93 | 0.66 | 0.65 | 1.31 | 0.90 | 0.56 | 0.64 | 1.28 |
| Top | 1.12 | 0.49 | 0.81 | 1.56 | 1.08 | 0.66 | 0.77 | 1.50 |
| **Province Fixed Effect** | Yes |  |  |  | Yes |  |  |  |

Note:

Model 1: ${Diabetes}_{i}=\beta_{0}+\beta_{1}{GLFE}_{i}+\beta_{2}{Covariates}_{i}+\lambda_{i}+\varepsilon_{i}\left( 1 \right)$

Model 2: ${Diabetes}_{i}=\beta_{0}+\beta_{1}{GLFE}_{i}+\beta_{2}{Migration Status}_{i}+\beta_{3}{Covariates}_{i}+\lambda_{i}+\varepsilon_{i}(2)$

**Supplement Table 6: Multivariable logistic regression results in Model 3 with all four urban-rural statuses.**

|  | **2011 Adolescence-Exposed vs.**  **2018 Childhood-Exposed (N=6,947)** | | | | **2011 Childhood-Exposed vs.**  **2018 Fetal-Exposed (N=6,840)** | | | | **2013 Fetal-Exposed vs.**  **2018 Non-Exposed (N=4,656)** | | | |
| --- | --- | --- | --- | --- | --- | --- | --- | --- | --- | --- | --- | --- |
|  | **Group 1** |  |  |  | **Group 2** |  |  |  | **Group 3** |  |  |  |
|  | **OR** | **P** | **95%** | **CI** | **OR** | **P** | **95%** | **CI** | **OR** | **P** | **95%** | **CI** |
| **Age** |  |  |  |  |  |  |  |  |  |  |  |  |
|  | 1.00 | 0.77 | 0.98 | 1.03 | 1.03 | 0.06 | 1.00 | 1.05 | 1.03 | 0.31 | 0.97 | 1.10 |
| **GLFF exposure** |  |  |  |  |  |  |  |  |  |  |  |  |
| Adolescence-exposed | 0.87 | 0.15 | 0.72 | 1.05 |  |  |  |  |  |  |  |  |
| Childhood-exposed | Ref |  |  |  | 1.07 | 0.51 | 0.87 | 1.32 |  |  |  |  |
| Fetal-exposed |  |  |  |  | Ref |  |  |  | 1.23 | 0.09 | 0.97 | 1.56 |
| Non-exposed |  |  |  |  |  |  |  |  | Ref |  |  |  |
| **Urban/Rural/migration Status** |  |  |  |  |  |  |  |  |  |  |  |  |
| Static urban residence | 1.84 | 0.00 | 1.44 | 2.34 | 1.48 | 0.03 | 1.03 | 2.14 | 1.38 | 0.10 | 0.94 | 2.04 |
| Rural-to-urban migration | 1.26 | 0.03 | 1.02 | 1.56 | 1.11 | 0.56 | 0.79 | 1.54 | 1.30 | 0.09 | 0.96 | 1.76 |
| Urban-to-rural migration | 1.55 | 0.12 | 0.89 | 2.70 | 1.24 | 0.74 | 0.35 | 4.37 | 1.77 | 0.26 | 0.65 | 4.83 |
| Static rural residence | Ref |  |  |  | Ref |  |  |  | Ref |  |  |  |
| **Adolescence-exposure X*** |  |  |  |  |  |  |  |  |  |  |  |  |
| Static urban residence | 1.05 | 0.80 | 0.73 | 1.52 |  |  |  |  |  |  |  |  |
| Rural-to-urban migration | 1.02 | 0.94 | 0.70 | 1.48 |  |  |  |  |  |  |  |  |
| Urban-to-rural migration | 0.92 | 0.83 | 0.41 | 2.05 |  |  |  |  |  |  |  |  |
| Static rural residence | Ref |  |  |  |  |  |  |  |  |  |  |  |
| **Childhood-exposure X*** |  |  |  |  |  |  |  |  |  |  |  |  |
| Static urban residence |  |  |  |  | 1.03 | 0.89 | 0.68 | 1.55 |  |  |  |  |
| Rural-to-urban migration |  |  |  |  | 1.05 | 0.80 | 0.72 | 1.54 |  |  |  |  |
| Urban-to-rural migration |  |  |  |  | 1.06 | 0.94 | 0.27 | 4.17 |  |  |  |  |
| Static rural residence |  |  |  |  | Ref |  |  |  |  |  |  |  |
| **Fetal-exposure X*** |  |  |  |  |  |  |  |  |  |  |  |  |
| Static urban residence |  |  |  |  |  |  |  |  | 1.04 | 0.88 | 0.63 | 1.72 |
| Rural-to-urban migration |  |  |  |  |  |  |  |  | 0.80 | 0.33 | 0.52 | 1.25 |
| Urban-to-rural migration |  |  |  |  |  |  |  |  | 0.93 | 0.93 | 0.21 | 4.13 |
| Static rural residence |  |  |  |  |  |  |  |  | Ref |  |  |  |
| **Sex** |  |  |  |  |  |  |  |  |  |  |  |  |
| Male | Ref |  |  |  | Ref |  |  |  | Ref |  |  |  |
| Female | 1.07 | 0.46 | 0.89 | 1.29 | 0.89 | 0.27 | 0.74 | 1.09 | 0.78 | 0.07 | 0.59 | 1.02 |
| **Education level** |  |  |  |  |  |  |  |  |  |  |  |  |
| No formal education | Ref |  |  |  | Ref |  |  |  | Ref |  |  |  |
| Below elementary school | 1.03 | 0.76 | 0.85 | 1.25 | 0.95 | 0.61 | 0.77 | 1.16 | 1.14 | 0.45 | 0.81 | 1.59 |
| Elementary school | 1.01 | 0.94 | 0.83 | 1.23 | 0.88 | 0.25 | 0.71 | 1.09 | 0.96 | 0.79 | 0.70 | 1.32 |
| Middle school | 1.04 | 0.75 | 0.83 | 1.31 | 0.98 | 0.85 | 0.78 | 1.23 | 1.10 | 0.53 | 0.81 | 1.49 |
| High school | 1.32 | 0.10 | 0.94 | 1.84 | 0.90 | 0.47 | 0.67 | 1.20 | 0.74 | 0.14 | 0.51 | 1.10 |
| College and above | 1.40 | 0.08 | 0.96 | 2.04 | 1.43 | 0.09 | 0.94 | 2.17 | 1.34 | 0.30 | 0.77 | 2.32 |
| **Marital status** |  |  |  |  |  |  |  |  |  |  |  |  |
| Unsingle | Ref |  |  |  | Ref |  |  |  | Ref |  |  |  |
| Single | 0.95 | 0.56 | 0.80 | 1.13 | 0.80 | 0.03 | 0.65 | 0.98 | 0.66 | 0.03 | 0.46 | 0.96 |
| **Self-reported health status** |  |  |  |  |  |  |  |  |  |  |  |  |
| Good | Ref |  |  |  | Ref |  |  |  | Ref |  |  |  |
| Fair | 1.98 | 0.00 | 1.43 | 2.75 | 1.89 | 0.00 | 1.38 | 2.57 | 2.10 | 0.00 | 1.46 | 3.02 |
| Poor | 3.95 | 0.00 | 2.84 | 5.50 | 3.70 | 0.00 | 2.70 | 5.07 | 4.70 | 0.00 | 3.22 | 6.86 |
| **Smoke status** |  |  |  |  |  |  |  |  |  |  |  |  |
| No | Ref |  |  |  | Ref |  |  |  | Ref |  |  |  |
| Yes | 0.74 | 0.00 | 0.62 | 0.87 | 0.66 | 0.00 | 0.55 | 0.79 | 0.79 | 0.08 | 0.61 | 1.03 |
| **Alcohol drink status** |  |  |  |  |  |  |  |  |  |  |  |  |
| No | Ref |  |  |  | Ref |  |  |  | Ref |  |  |  |
| Yes | 0.87 | 0.07 | 0.75 | 1.01 | 0.89 | 0.12 | 0.76 | 1.03 | 0.84 | 0.12 | 0.68 | 1.04 |
| **Guardians alcohol/drug issue** |  |  |  |  |  |  |  |  |  |  |  |  |
| No | Ref |  |  |  | Ref |  |  |  | Ref |  |  |  |
| Yes | 1.29 | 0.05 | 1.00 | 1.65 | 1.62 | 0.00 | 1.26 | 2.08 | 1.48 | 0.02 | 1.08 | 2.03 |
| **Self-report childhood health** |  |  |  |  |  |  |  |  |  |  |  |  |
| Better | Ref |  |  |  | Ref |  |  |  | Ref |  |  |  |
| Same | 0.98 | 0.78 | 0.85 | 1.13 | 0.91 | 0.21 | 0.79 | 1.05 | 0.81 | 0.04 | 0.67 | 0.99 |
| Worse | 1.07 | 0.52 | 0.87 | 1.31 | 0.84 | 0.11 | 0.67 | 1.04 | 0.79 | 0.11 | 0.59 | 1.06 |
| **Self-report childhood finance** |  |  |  |  |  |  |  |  |  |  |  |  |
| Better | Ref |  |  |  | Ref |  |  |  | Ref |  |  |  |
| Same | 0.94 | 0.63 | 0.74 | 1.20 | 0.87 | 0.28 | 0.69 | 1.12 | 0.74 | 0.05 | 0.55 | 1.01 |
| Worse | 1.00 | 0.98 | 0.78 | 1.28 | 0.94 | 0.64 | 0.73 | 1.21 | 0.73 | 0.05 | 0.53 | 1.00 |
| **Employment Status** |  |  |  |  |  |  |  |  |  |  |  |  |
| Agriculture | Ref |  |  |  | Ref |  |  |  | Ref |  |  |  |
| Non-agriculture | 0.79 | 0.06 | 0.62 | 1.01 | 0.83 | 0.12 | 0.66 | 1.05 | 0.87 | 0.35 | 0.65 | 1.16 |
| Retired or unemployment | 1.21 | 0.05 | 1.00 | 1.45 | 1.24 | 0.03 | 1.02 | 1.50 | 1.17 | 0.25 | 0.89 | 1.54 |
| **Health insurance** |  |  |  |  |  |  |  |  |  |  |  |  |
| No | Ref |  |  |  | Ref |  |  |  | Ref |  |  |  |
| Yes | 1.21 | 0.05 | 1.00 | 1.47 | 1.11 | 0.32 | 0.90 | 1.37 | 1.24 | 0.14 | 0.93 | 1.65 |
| **Income level** |  |  |  |  |  |  |  |  |  |  |  |  |
| Bottom | Ref |  |  |  | Ref |  |  |  | Ref |  |  |  |
| Middle | 1.01 | 0.91 | 0.82 | 1.24 | 1.01 | 0.96 | 0.80 | 1.26 | 0.90 | 0.55 | 0.63 | 1.28 |
| Top | 1.12 | 0.26 | 0.92 | 1.37 | 1.17 | 0.16 | 0.94 | 1.46 | 1.08 | 0.67 | 0.77 | 1.50 |
| **Province Fixed Effect** | **Yes** |  |  |  | **Yes** |  |  |  | **Yes** |  |  |  |

Note:

Model 3: ${Diabetes}_{i}=\beta_{0}+\beta_{1}{GLFE}_{i}+\beta_{2}{Migration Status}_{i}+\beta_{3}{GLFE}_{i}\times{Migration Status}_{i}+\beta_{4}{Covariates}_{i}+\lambda_{i}+\varepsilon_{i}(3)$

**Supplement Table 7: Multivariable Logistic Regression of Diabetes Risk in Group 3 (Age 50-56)**

|  | **2013 Fetal-exposed vs. 2018 Non-exposed** | | | | | | | | | | | |
| --- | --- | --- | --- | --- | --- | --- | --- | --- | --- | --- | --- | --- |
| **N=5,265** | **OR** | **P** | **95%** | **CI** | **OR** | **P** | **95%** | **CI** | **OR** | **P** | **95%** | **CI** |
| **GLFF exposure** |  |  |  |  |  |  |  |  |  |  |  |  |
| Non-exposed | Ref |  |  |  | Ref |  |  |  | Ref |  |  |  |
| Fetal-exposed | 1.203 | 0.035 | 1.013 | 1.430 | 1.194 | 0.044 | 1.005 | 1.420 | 1.261 | 0.043 | 1.007 | 1.579 |
| **Urbanicity** |  |  |  |  |  |  |  |  |  |  |  |  |
| Static urban residence |  |  |  |  | 1.489 | 0.003 | 1.141 | 1.942 | 1.507 | 0.021 | 1.063 | 2.137 |
| Rural-to-urban migration |  |  |  |  | 1.244 | 0.054 | 0.996 | 1.554 | 1.360 | 0.035 | 1.022 | 1.811 |
| Urban-to-rural migration |  |  |  |  | 1.293 | 0.502 | 0.611 | 2.738 | 1.408 | 0.495 | 0.527 | 3.764 |
| Static rural residence |  |  |  |  | Ref |  |  |  | Ref |  |  |  |
| **Adolescence-exposed X** |  |  |  |  |  |  |  |  |  |  |  |  |
| Static urban residence |  |  |  |  |  |  |  |  | 0.971 | 0.898 | 0.619 | 1.523 |
| Rural-to-urban migration |  |  |  |  |  |  |  |  | 0.817 | 0.338 | 0.539 | 1.236 |
| Urban-to-rural migration |  |  |  |  |  |  |  |  | 0.828 | 0.801 | 0.191 | 3.590 |
| Static rural residence |  |  |  |  |  |  |  |  | Ref |  |  |  |
| **Sex** |  |  |  |  |  |  |  |  |  |  |  |  |
| Male | Ref |  |  |  | Ref |  |  |  | Ref |  |  |  |
| Female | 0.776 | 0.050 | 0.602 | 1.000 | 0.763 | 0.037 | 0.592 | 0.983 | 0.761 | 0.035 | 0.590 | 0.981 |
| **Education level** |  |  |  |  |  |  |  |  |  |  |  |  |
| No formal education | Ref |  |  |  | Ref |  |  |  | Ref |  |  |  |
| Below elementary school | 1.115 | 0.496 | 0.816 | 1.524 | 1.097 | 0.564 | 0.802 | 1.500 | 1.102 | 0.544 | 0.805 | 1.508 |
| Elementary school | 0.928 | 0.626 | 0.688 | 1.253 | 0.900 | 0.493 | 0.666 | 1.216 | 0.901 | 0.498 | 0.667 | 1.218 |
| Middle school | 1.125 | 0.410 | 0.850 | 1.489 | 1.058 | 0.696 | 0.797 | 1.405 | 1.058 | 0.698 | 0.797 | 1.405 |
| High school | 0.874 | 0.447 | 0.619 | 1.235 | 0.777 | 0.163 | 0.545 | 1.108 | 0.775 | 0.159 | 0.543 | 1.105 |
| College and above | 1.879 | 0.006 | 1.198 | 2.947 | 1.492 | 0.103 | 0.922 | 2.415 | 1.494 | 0.103 | 0.922 | 2.421 |
| **Marital status** |  |  |  |  |  |  |  |  |  |  |  |  |
| Unsingle | Ref |  |  |  | Ref |  |  |  | Ref |  |  |  |
| Single | 0.622 | 0.008 | 0.437 | 0.884 | 0.604 | 0.005 | 0.424 | 0.860 | 0.605 | 0.005 | 0.425 | 0.861 |
| **Self-reported health status** |  |  |  |  |  |  |  |  |  |  |  |  |
| Good | Ref |  |  |  | Ref |  |  |  | Ref |  |  |  |
| Fair | 1.894 | 0.000 | 1.368 | 2.622 | 1.926 | 0.000 | 1.391 | 2.668 | 1.936 | 0.000 | 1.397 | 2.682 |
| Poor | 4.185 | 0.000 | 2.995 | 5.848 | 4.368 | 0.000 | 3.121 | 6.113 | 4.375 | 0.000 | 3.125 | 6.124 |
| **Smoke status** |  |  |  |  |  |  |  |  |  |  |  |  |
| No | Ref |  |  |  | Ref |  |  |  | Ref |  |  |  |
| Yes | 0.814 | 0.091 | 0.641 | 1.033 | 0.818 | 0.099 | 0.644 | 1.038 | 0.817 | 0.098 | 0.644 | 1.038 |
| **Drink status** |  |  |  |  |  |  |  |  |  |  |  |  |
| No | Ref |  |  |  | Ref |  |  |  | Ref |  |  |  |
| Yes | 0.857 | 0.125 | 0.703 | 1.044 | 0.855 | 0.120 | 0.701 | 1.042 | 0.854 | 0.119 | 0.701 | 1.041 |
| **Employment Status** |  |  |  |  |  |  |  |  |  |  |  |  |
| Agriculture | Ref |  |  |  | Ref |  |  |  | Ref |  |  |  |
| Non-agriculture | 0.874 | 0.339 | 0.664 | 1.151 | 0.830 | 0.188 | 0.628 | 1.096 | 0.831 | 0.192 | 0.629 | 1.098 |
| No job | 1.210 | 0.144 | 0.937 | 1.562 | 1.119 | 0.397 | 0.862 | 1.453 | 1.123 | 0.382 | 0.865 | 1.459 |
| **Health insurance** |  |  |  |  |  |  |  |  |  |  |  |  |
| No | Ref |  |  |  | Ref |  |  |  | Ref |  |  |  |
| Yes | 1.195 | 0.188 | 0.916 | 1.558 | 1.217 | 0.148 | 0.933 | 1.587 | 1.220 | 0.143 | 0.935 | 1.593 |
| **Income level** |  |  |  |  |  |  |  |  |  |  |  |  |
| Bottom | Ref |  |  |  | Ref |  |  |  | Ref |  |  |  |
| Middle | 0.969 | 0.852 | 0.698 | 1.346 | 0.941 | 0.717 | 0.677 | 1.308 | 0.939 | 0.706 | 0.675 | 1.305 |
| Top | 1.199 | 0.248 | 0.881 | 1.631 | 1.144 | 0.394 | 0.840 | 1.559 | 1.144 | 0.395 | 0.839 | 1.559 |
| **Province Fixed Effect** | Yes |  |  |  | Yes |  |  |  | Yes |  |  |  |

**Supplement Table 8:** **Comparison of diabetes risk between different urban-rural statuses conditioning on GLFF exposure statuses across the three age groups**

| **2011 Adolescence-exposed vs. 2018 Childhood-exposed** | | | |
| --- | --- | --- | --- |
| **Static rural residence vs.** | **Diff** | **95%** | **CI** |
| **Static urban residence** |  |  |  |
| Childhood-exposed | 0.081 | 0.045 | 0.118 |
| Adolescence-exposed | 0.091 | 0.046 | 0.137 |
| **Rural-to-urban migration** |  |  |  |
| Childhood-exposed | 0.031 | 0.004 | 0.059 |
| Adolescence-exposed | 0.031 | -0.008 | 0.069 |
| **Urban-to-rural migration** |  |  |  |
| Childhood-exposed | 0.062 | -0.022 | 0.146 |
| Adolescence-exposed | 0.051 | -0.032 | 0.133 |
| **2011 Childhood-exposed vs. 2018 Fetal-exposed** | | |  |
| **Static rural residence vs.** | **Diff** | **95%** | **CI** |
| **Static urban residence** |  |  |  |
| Fetal-exposed | 0.046 | -0.002 | 0.093 |
| Childhood-exposed | 0.053 | 0.021 | 0.086 |
| **Rural-to-urban migration** |  |  |  |
| Fetal-exposed | 0.008 | -0.030 | 0.047 |
| Childhood-exposed | 0.021 | -0.005 | 0.047 |
| **Urban-to-rural migration** |  |  |  |
| Fetal-exposed | -0.015 | -0.150 | 0.120 |
| Childhood-exposed | 0.046 | -0.033 | 0.124 |
| **2013 Fetal-exposed vs. 2018 Non-exposed** | | |  |
| **Static rural residence vs.** | **Diff** | **95%** | **CI** |
| **Static urban residence** |  |  |  |
| Non-exposed | 0.043 | 0.003 | 0.084 |
| Fetal-exposed | 0.046 | 0.001 | 0.092 |
| **Rural-to-urban migration** |  |  |  |
| Non-exposed | 0.031 | 0.001 | 0.062 |
| Fetal-exposed | 0.012 | -0.025 | 0.048 |
| **Urban-to-rural migration** |  |  |  |
| Non-exposed | 0.035 | -0.078 | 0.149 |
| Fetal-exposed | 0.017 | -0.116 | 0.151 |

**Supplement Table 9: Comparison of diabetes risk between different GLFF exposure statuses conditioning on urban-rural statuses across the three age groups**

| **2011 Adolescence-exposed vs. 2018 Childhood-exposed** | | | |
| --- | --- | --- | --- |
|  | **Diff** | **95%** | **CI** |
| Static urban residence | -0.009 | -0.057 | 0.040 |
| Rural-to-urban migration | -0.019 | -0.061 | 0.022 |
| Urban-to-rural migration | -0.030 | -0.145 | 0.084 |
| Static rural residence | -0.018 | -0.040 | 0.003 |
| **2011 Childhood-exposed vs. 2018 Fetal-exposed** | | | |
|  | **Diff** | **95%** | **CI** |
| Static urban residence | 0.008 | -0.041 | 0.056 |
| Rural-to-urban migration | 0.013 | -0.026 | 0.052 |
| Urban-to-rural migration | 0.061 | -0.092 | 0.214 |
| Static rural residence | 0.000 | -0.023 | 0.023 |
| **2013 Fetal-exposed vs. 2018 Non-exposed** | | |  |
|  | **Diff** | **95%** | **CI** |
| Static urban residence | 0.026 | -0.025 | 0.077 |
| Rural-to-urban migration | 0.003 | -0.037 | 0.044 |
| Urban-to-rural migration | 0.005 | -0.166 | 0.176 |
| Static rural residence | 0.023 | 0.000 | 0.046 |
